# Ketamine versus etomidate for rapid sequence intubation in patients with trauma: a retrospective study in a level 1 trauma center in Korea

**DOI:** 10.1186/s12873-023-00833-7

**Published:** 2023-05-29

**Authors:** Jinjoo Kim, Kyoungwon Jung, Jonghwan Moon, Junsik Kwon, Byung Hee Kang, Jayoung Yoo, Seoyoung Song, Eunsook Bang, Sora Kim, Yo Huh

**Affiliations:** 1 Division of Trauma Surgery, Department of Surgery, Ajou University School of Medicine, Suwon, Republic of Korea; 2 Gyeonggi Southern Regional Trauma Centre, Ajou University Hospital, Suwon, Republic of Korea

**Keywords:** Ketamine, Etomidate, Rapid sequence intubation, Trauma, Resuscitation

## Abstract

**Background:**

Ketamine and etomidate are commonly used as sedatives in rapid sequence intubation (RSI). However, there is no consensus on which agent should be favored when treating patients with trauma. This study aimed to compare the effects of ketamine and etomidate on first-pass success and outcomes of patients with trauma after RSI-facilitated emergency intubation.

**Methods:**

We retrospectively reviewed 944 patients who underwent endotracheal intubation in a trauma bay at a Korean level 1 trauma center between January 2019 and December 2021. Outcomes were compared between the ketamine and etomidate groups after propensity score matching to balance the overall distribution between the two groups.

**Results:**

In total, 620 patients were included in the analysis, of which 118 (19.9%) were administered ketamine and the remaining 502 (80.1%) were treated with etomidate. Patients in the ketamine group showed a significantly faster initial heart rate (105.0 ± 25.7 vs. 97.7 ± 23.6, *p = 0.003*), were more hypotensive (114.2 ± 32.8 mmHg vs. 139.3 ± 34.4 mmHg, *p < 0.001*), and had higher Glasgow Coma Scale (9.1 ± 4.0 vs. 8.2 ± 4.0, *p = 0.031*) and Injury Severity Score (32.5 ± 16.3 vs. 27.0 ± 13.3, *p < 0.001*) than those in the etomidate group. There were no significant differences in the first-pass success rate (90.7% vs. 90.1%, *p > 0.999)*, final mortality (16.1% vs. 20.6, *p = 0.348)*, length of stay in the intensive care unit (days) (8 [4, 15] (Interquartile range)), vs. 10 [4, 21], *p = 0.998*), ventilator days (4 [2, 10] vs. 5 [2, 13], *p = 0.735*), and hospital stay (days) (24.5 [10.25, 38.5] vs. 22 [8, 40], *p = 0.322*) in the 1:3 propensity score matching analysis.

**Conclusion:**

In this retrospective study of trauma resuscitation, those receiving intubation with ketamine had greater hemodynamic instability than those receiving etomidate. However, there was no significant difference in clinical outcomes between patients sedated with ketamine and those treated with etomidate.

**Supplementary Information:**

The online version contains supplementary material available at 10.1186/s12873-023-00833-7.

## Background

Rapid sequence intubation (RSI) is the recommended procedure for facilitating emergency orotracheal intubation in patients with trauma. Considering the risks of aspiration, this emergent technique has been widely accepted as an important strategy for unprepared patients [1]. Ideally, this technique could quickly promote optimal intubation conditions by increasing the first-pass intubation rate while minimizing adverse events, such as hemodynamic changes, in severely injured patients. Several induction agents are available for RSI; however, there is no consensus regarding which agent should be favored for severely injured patients [2].

Among induction agents, ketamine and etomidate are commonly used to sedate patients during emergency tracheal intubation in the emergency department (ED) or intensive care unit (ICU) [3–5]. In terms of sedatives, both agents are well-known for their short duration of action and relatively rapid onset with a good hemodynamic profile [6]. In Advanced Trauma Life Support, etomidate is recommended as an induction drug for patients with trauma in whom post-intubation hypotension (PIH) could be associated with adverse outcomes; [7] however, etomidate can cause reversible adrenal insufficiency (AI). Furthermore, although AI is associated with increased mortality and morbidity among critically ill patients, its clinical significance after etomidate administration has not yet been confirmed [8–11].

Ketamine is not associated with adrenal suppression and is known to stimulate the sympathetic nervous system as well as catecholamine release, which could increase the heart rate and blood pressure by exerting beneficial effects in hemodynamically unstable trauma patients [12, 13]. Therefore, ketamine could be used as an alternative to etomidate, with the benefits of hemodynamic stability and adrenal function. In contrast, catecholamine release can cause myocardial depression and is related to cardiac arrest during emergency endotracheal intubation [14, 15]. We hypothesized that induction agents could affect clinical outcomes of trauma patients.

Therefore, the primary outcome of this study, aimed at comparing the first-pass rate and secondary outcomes are the outcomes of patients with trauma including final mortality, hospital day, Intensive Care Unit (ICU) Length of stay(LOS), ventilator day after RSI-facilitated emergency intubation using either etomidate or ketamine.

This study, therefore, aimed to compare the first-pass rate and outcomes of patients with trauma after RSI-facilitated emergency intubation using either etomidate or ketamine.

## Methods

Our institution is a tertiary academic hospital located in Suwon, Gyeonggi province, Korea, a city with a population of 1.3 million. It operates the southern Gyeonggi level 1 trauma center. The trauma center building is an independent facility with 100 units, including 2 trauma bays, 40 trauma ICUs, and 3 trauma operation theatres. Further, according to the Korean trauma data bank, the trauma center admits approximately 3000 acutely injured patients annually.

Induction agents and intubation techniques were selected at the discretion of the trauma team, which included well-trained and dedicated trauma general surgeons, cardiothoracic surgeons, and emergency medicine specialists. Our trauma bays are furnished with basic and advanced airway equipment, such as conventional laryngoscopes, video laryngoscopes (both Glidescopes® and C-MAC®), and suction equipment, as well as vasopressors, induction agents, and neuromuscular blockers. The decision to intubate patients was made by the trauma team leader based on a combination of clinical signs and risk–benefit assessment.

Based on the Eastern Association for the Surgery of Trauma practice management guidelines, our team routinely uses RSI techniques that align with the generally accepted approach to emergency intubation for severe injured patients [1]. This includes experienced operators, pulse-oximetry monitoring, cervical immobilization, routine use of RSI with adequate sedation and neuromuscular blockade, video-laryngoscopy for higher intubation success rate, confirmation of endotracheal tube placement using end-tidal CO_2_ detection, as well as maintenance of adequate oxygenation and hemodynamic stability during the endotracheal intubation period [17]. RSI induction was delivered with either etomidate or ketamine within the suggested dose ranges (ketamine 1–2 mg/kg IV: etomidate 0.2–0.3 mg/kg/IV).

**Fig. 1:**
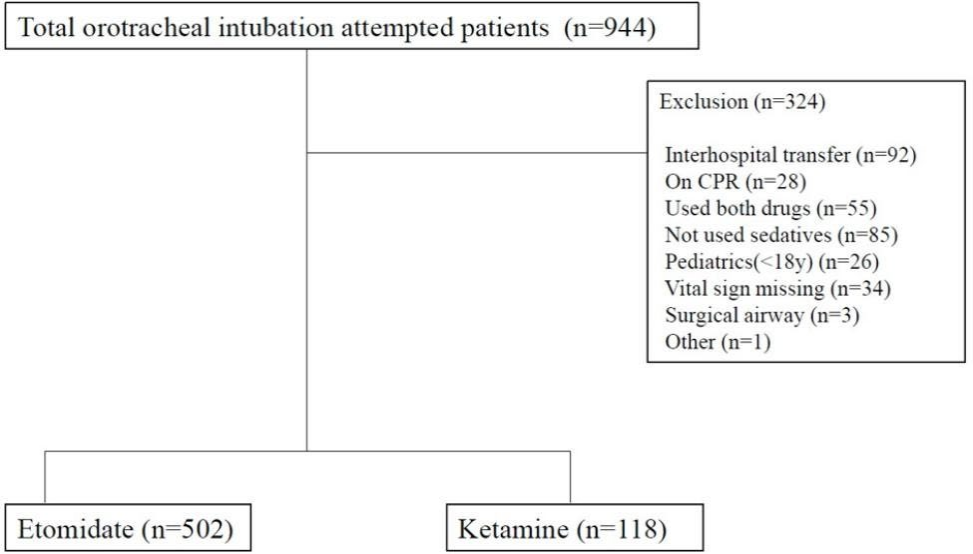
Flowchart for the selection of study population. CPR: cardiopulmonary resuscitation

All adult patients, aged ≥ 18 years, with trauma who needed emergency endotracheal intubation using either ketamine or etomidate in the trauma bays of our institution between January 2019 and December 2021 were included. Pediatric patients (< 18 years), pregnant women, as well as patients with cardiac arrest on arrival, expired patients during resuscitation in the trauma bay, Do-Not-Resuscitate orders, and missing data, were excluded from the study. Additionally, we excluded patients who had received no induction agent or agents other than etomidate or ketamine, underwent cricothyroidotomy, or had been transferred from other hospitals. (Fig. 1) The authors reviewed electronic medical records of patients included in this study. Demographic information, including age, sex, and mechanism of injury, was reviewed. Injury severity was evaluated using the shock index, initial Glasgow Coma Scale (GCS) score, Anatomic Injury Score (AIS), and Injury Severity Score (ISS). Continuous variables were analyzed using Student’s *t*-test and are presented as mean ± standard deviation and median Inter-quartile range, (IQR). Categorical variables were analyzed using the chi-square test and are expressed as proportions. Propensity score matching (PSM) was used to minimize selection bias. A 1:3 PSM was performed to adjust for injury severity and confounding baseline characteristics. Propensity scores were estimated using age, sex, mechanism of injury, shock index, GCS, and ISS. Standardized differences of less than 0.2 indicated a good balance between the two groups for a given covariate. After matching, the chi-square test and Student’s *t*-test were used for the analysis. All statistical analyses were performed using R software, version 4.0.5. A *p-*value < 0.05 was considered significant. Finally, power and sample size were calculated retrospectively using G Power 3.1.3 software.

### Ethics statement

The need for obtaining informed consent was waived by the Institutional Review Board (IRB) of Ajou University Hospital (IRB No. AJOUIRB-DB-2022-353) because of the observational nature of the study. This study is reported in accordance with the Strengthening the Reporting of Observational Studies in Epidemiology (STROBE) guidelines for observational studies [16.]

## Results

In total, 620 patients were included in the analysis, of which 118 (19.9%) were administered ketamine and 502 (80.1%) were treated with etomidate (Table 1). The patients who received ketamine had lower initial blood pressure than the patients who received etomidate. (114.2 ± 32.8 vs. 139.3 ± 34.4, *p < 0.001) and* significantly faster initial heart rate (105.0 ± 25.7 vs. 97.7 ± 23.6, *p = 0.003*), and had higher GCS (9.1 ± 4.0 vs. 8.2 ± 4.0, *p = 0.031*) and ISS (32.5 ± 16.3 vs. 27.0 ± 13.3, *p < 0.001*) than those in the etomidate group. Ketamine was used more frequently in patients with severe chest, abdominal, and pelvic injuries than in patients with severe head injuries (Table 1).

**Table 1:** Baseline characteristics of trauma patients before and after propensity matching

	Unmatched patients				1:3 propensity score matched patients		
Variables	Ketamine	Etomidate	*p-value*	Ketamine		Etomidate	*p-value*
	(n = 118)	(n = 502)		(n = 118)		(n = 354)	
Age, mean ± SD (years)	50.0 ± 18.8	50.8 ± 17.8	*0.64*	50.0 ± 18.8		50.6 ± 18.0	*0.74*
Sex, n (%)			*0.338*				*0.682*
Male	94 (79.7)	421 (83.9)		94 (79.7)		290 (81.9))	
Mechanism of injury, n (%)			*0.563*				*0.953*
Free fall	36 (30.5)	128 (25.5)		36 (30.5)		96 (27.1)	
Motor vehicle accident	55 (46.6)	250 (50.6)		55 (46.6)		167 (47.2)	
Ground fall	2 (1.7)	18 (3.6)		2 (1.7)		6 (1.7)	
Other blunt trauma	4 (3.4)	22 (4.4)		4 (3.4)		14 (4.0)	
Penetrating injury	15 (12.7)	47 (9.4)		15 (12.7)		45 (12.7)	
Unknown	6 (5.1)	33 (6.6)		6 (5.1)		26 (7.3)	
Initial HR, mean ± SD (beats/min)	105.0 ± 25.7	97.7 ± 23.6	*0.003*	105.0 ± 25.7		99.1 ± 24.8	*0.029*
Initial SBP, mean ± SD (mmHg)	114.2 ± 32.8	139.3 ± 34.4	*< 0.001*	114.2 ± 32.8		136.1 ± 34.7	*< 0.001*
Initial SBP, n (%)			*< 0.001*				*< 0.001*
≤ 90 mmHg	27 (22.9)	38 (7.6)		27 (22.9)		34 (9.6)	
Shock index, mean ± SD	1.5 ± 0.6	1.4 ± 0.5	*0.502*	1.5 ± 0.6		1.5 ± 0.6	*0.591*
Shock index, n (%)			*> 0.999*				*> 0.999*
≤ 0.9	18 (15.3)	76 (15.1)		18 (15.3)		54 (14.7)	
Initial GCS, mean ± SD	9.1 ± 4.0	8.2 ± 4.0	*0.031*	9.1 ± 4.0		8.7 ± 4.1	*0.358*
ISS, mean ± SD	32.5 ± 16.3	27.0 ± 13.3	*< 0.001*	32.5 ± 16.3		28.0 ± 13.3	*0.101*
ISS, n (%)			*0.084*				*0.65*
>15	108 (87.3)	401 (79.9)		108 (87.3)		301 (85.0)	

HR, heart rate; SD, standard deviation; SBP, systolic blood pressure; MAP, mean arterial pressure; GCS, Glasgow Coma Scale; ISS, Injury Severity Scale

The first-pass success rate between the unmatching two groups, there is no difference (90.7% vs. 89.2%, *p* = 0.771), and after 1:3 PSM analysis, there were no significant difference in the first-pass success rate (90.7% vs. 90.1%, *p* > 0.999). Furthermore, between unmatching two groups, final mortality (16.1% vs. 19.5%, *p* = 0.468), ICU LOS (8 days (IQR) [4, 15] vs. 8 days [3, 19], *p* = 0.783), ventilator days (4 days [2, 10] vs. 4 days [2, 13], *p* = 0.964), and hospital stay (24.5 days [10.25, 38.5] vs. 19 [7, 38] days, *p* = 0.187) are no significant difference. Likewise, after 1:3 PSM analysis, final mortality (16.1% vs. 20.6%, *p* = 0.348), ICU LOS (8 days [4, 15] vs. 10 days [4,21], *p* = 0.998), ventilator days (4 days [2, 10] vs. 5 days [2, 13], *p* = 0.735) and hospital stay (24.5 days [10.25, 38.5] vs. 22 days [8, 40], *p* = 0.322) were no difference between two groups (Table 2).

**Table 2:** Outcomes of administering ketamine or etomidate before and after propensity matching

		Unmatched patients				1:3 propensity score matching patients		
**Outcomes**	**Ketamine** **(n = 118)**		**Etomidate (n = 502)**	**95% CI**	**Ketamine** **(n = 118)**		**Etomidate (n = 354)**	**95% CI**
First-pass success rate, n (%)	107 (90.7)		448 (89.2)	(-0.13, 0.08)	107 (90.7)		319 (90.1)	(-0.15, 0.13)
Mortality, n (%)	19 (16.1)		98 (19.5)	(-0.05, 0.12)	19 (16.1)		73 (20.6)	(-0.05, 0.15)
ICU LOS, days, median (IQR)	8 (4–15)		8 (3–19)	(-5.13, 6.79)	8 (4–15)		10 (4–21)	(-6.01, 6.03)
Ventilator days, median (IQR)	4 (2–10)		4 (2–13)	(-3.5, 3.34)	4 (2–10)		5 (2–13)	(-4.1, 2.9)
Hospital LOS, days, median (IQR)	24.5 (10.25–38.5)		19 (7–38)	(-2.41, 12.23)	24.5 (10.25–38.5)		22 (8–40)	(-3.69, 11.15)

CI, Confidence Interval; ICU, intensive care unit; LOS, length of stay; IQR, Interquartile range

## Discussion

In the current study, there were no significant differences in the first-pass intubation success and final mortality rates between the ketamine and etomidate groups. However, we found that trauma physicians tended to choose ketamine for patients with hypovolemic shock, considering the lack of propensity-matched results.

Propofol, like etomidate, is a commonly used medication for RSI. However, it is associated with hypotension from systemic vasodilation and direct myocardial depression, which could be inappropriate for use in patients with hypovolemic shock [5, 17]. Etomidate has been considered the agent of choice for RSI because of several advantages, including a favorable hemodynamic profile, protection from cerebral and myocardial ischemia, as well as minimal histamine release [8]. In contrast, the impact of etomidate is related to AI in patients with trauma via the inhibition of 11*β*-hydroxylase and a decrease in adrenocortical function [9, 18]. Ketamine is a well-known, non-competitive *N*-methyl-D-aspartate receptor antagonist, introduced as an anesthetic in 1964. Owing to its analgesic properties, rapid onset of action, and respiratory stability, ketamine is widely used in procedural sedation, especially for painful procedures. Historically, ketamine has been reluctantly used in patients with suspected traumatic brain injury due to concerns related to intracranial pressure (ICP) [19]. Furthermore, ketamine is also known to be a direct myocardial depressant and could cause hypotension in patients with catecholamine depletion [14, 15]. Therefore, ketamine has not been deemed an attractive induction agent for severely injured patients, especially those with traumatic brain injury. However, this agent is known to increase blood pressure and heart rate through sympathetic nervous system stimulation, which is potentially appropriate for hemodynamically unstable and acutely ill patients. [20] Recently, the use of ketamine has been increasing owing to its safety, hemodynamic profile, and effectiveness in the prehospital setting and ED [13]. In this context, we found that patients in the ketamine group tended to have higher GCS and lower head AIS than those in the etomidate group; they also showed more features of hemodynamic instability on arrival to the trauma bay prior to induction for endotracheal intubation. For this reason, several studies have been conducted to compare ketamine with other induction agents for RSI. Matchet et al. reported that critically ill patients, including patients with trauma, randomized to ketamine and etomidate showed higher 7-day survival; however, there were no differences in 28-day mortality, mechanical ventilator days, and changes in Sequential Organ Failure Assessment scores between the ketamine and etomidate groups [4]. A study by Breindahl et al. reported no significant differences in mortality between ketamine and propofol groups [13]. In this study, among patients with trauma intubated using the RSI technique, we found no differences in final mortality, ICU LOS, and ventilator days between the ketamine and etomidate groups before and after PSM.

Our study has several limitations owing to the retrospective nature of the data. First, this was a single-center retrospective study; therefore, the generalizability of the results may be limited. The number of patients who were administered etomidate was an absolute majority in this study, and there could be potential selection bias in this study population. Although we analyzed data after 1:3 PSM to minimize bias, a residual confounding effect cannot be ruled out. Second, it would be difficult to explain why physicians chose specific induction agents due to the retrospective nature of the data analysis. Third, the current study excluded patients who died in trauma bays during resuscitation. Fourth, our team, which consists of well-trained trauma surgeons and emergency medicine specialists, routinely uses advanced techniques and practices, such as neuromuscular blockade and video-laryngoscopy, for emergency intubation in the trauma bay. Thus, the first-pass success rate was relatively higher than that in other studies [3]. Furthermore, because not all hospitals have these advanced resources, our protocol itself limits the generalizability of our findings. Fifth, we could not analyze PIH, and the incidence of AI associated with etomidate administration. In addition, we could not conduct a study on ICP elevation after ketamine administration. Therefore, further prospective studies are warranted to prove several adverse effects of induction agents in trauma patients. Finally, the long-term outcomes were not reported in this study, making it difficult to evaluate any important outcomes other than in-hospital results. However, this study is one of the largest studies comparing ketamine and etomidate for RSI in the trauma population, and we found no difference in first pass intubation success or clinical outcomes in patients with hemorrhagic shock. Although the use of etomidate is nearly universal, ketamine is being advocated for RSI in the ED or prehospital stage.

## Conclusion

In this retrospective study of patients with traumatic shock who received ketamine or etomidate for RSI, there were no differences in first pass intubation success or outcomes of final mortality, ventilator days, or hospital length of stay.

## Electronic supplementary material

Below is the link to the electronic supplementary material.

Supplementary Material 1

## Data Availability

The datasets used and/or analyzed during the current study are available from the corresponding author upon reasonable request.

## Abbreviations

RSI: Rapid sequence intubation
ED: Emergency department
ICU: Intensive care unit
LOS: Length of stay
PIH: Post-intubation hypotension
AI: Adrenal insufficiency
GCS: Glasgow Coma Scale
AIS: Anatomic Injury Score
ISS: Injury Severity Score
PSM: Propensity score matching
ICP: Intracranial pressure
IQR: Interquartile range
