# Supplementary material for: Ketamine versus etomidate for rapid sequence intubation in patients with trauma: a retrospective study in a level 1 trauma center in Korea
Source: BMC Emerg Med. 2023 May 29;23:57. doi: 10.1186/s12873-023-00833-7 (PMC10228132; doi:10.1186/s12873-023-00833-7)
Supplement: Supplementary file 1 — Supplementary Material 1 [file 12873_2023_833_MOESM1_ESM.docx]

**Supplementary Material**

Table A

Comparing AIS between Ketamine group and Etomidate group and associated injuries

|  | **Unmatched patients** | | | | **1:3 propensity score matched patients** | | |
| --- | --- | --- | --- | --- | --- | --- | --- |
| **Variables** | **Ketamine** | **Etomidate** | ***p-value*** | **Ketamine** | | **Etomidate** | ***p-value*** |
|  | **(n=118)** | **(n=502)** |  | **(n=118)** | | **(n=354)** |  |
| AIS, mean ± SD |  |  |  |  | |  |  |
| Head and neck | 2.3 ± 1.9 | 2.9 ± 2.0 | *0.004* | 2.28 ± 1.87 | | 2.97 ± 2.0 | *0.001* |
| Face | 0.6 ± 1.0 | 0.7 ± 0.9 | *0.402* | 0.59 ± 0.9 | | 0.72 ± 0.94 | *0.208* |
| Chest | 2.9 ± 1.5 | 2.4 ± 1.4 | *< 0.001* | 2.87 ± 1.5 | | 2.53 ± 1.3 | *0.019* |
| Abdomen | 1.7 ± 1.7 | 0.9 ± 1.4 | *< 0.001* | 1.73 ± 1.68 | | 1.06 ± 1.49 | *<0.001* |
| Pelvis and extremities | 1.9 ± 1.9 | 1.5 ± 1.6 | *0.012* | 1.92 ± 1.85 | | 1.7 ± 1.62 | *0.206* |
| External injuries | 0.7 ± 0.6 | 0.8 ± 0.5 | *0.479* | 0.7 ± 0.62 | | 0.75 ± 0.54 | *0.474* |
| Known associated injuries, n (%) |  |  |  |  | |  |  |
| Head and neck | 78 (66.1) | 373 (74.3) | *0.092* | 78 (66.1) | | 263 (74.3) | *0.019* |
| Face | 38 (32.2) | 194 (38.7) | *0.232* | 38 (32.2) | | 144 (60.7) | *0.126* |
| Chest | 99 (83.9) | 392 (78.1) | *0.203* | 99 (83.9) | | 289 (81.6) | *0.677* |
| Abdomen | 68 (57.6) | 158 (31.5) | *< 0.001* | 68 (57.6) | | 132 (37.3) | *<0.001* |
| Pelvis and extremities | 71 (60.2) | 268 (53.4) | *0.219* | 71 (60.2) | | 214 (60.5) | *>0.999* |
| External injuries | 73 (61.9) | 355 (70.7) | *0.078* | 73 (61.9) | | 248 (70.1) | *0.124* |

AIS, Abbreviated Injury Scale; SD, standard deviation
